# Discussion on the mechanism of Danggui Sini decoction in treating diabetic foot based on network pharmacology and molecular docking and verification of the curative effect by meta-analysis

**DOI:** 10.3389/fendo.2024.1347021

**Published:** 2024-02-23

**Authors:** Xiyu Ni, Huhe Bao, Jiaxing Guo, Deer Li, Lihang Wang, Wanyin Zhang, Guanwen Sun

**Affiliations:** ^1^ Graduate School, Inner Mongolia Medical University, Hohhot, China; ^2^ Department of Traumatology and Orthopedics, Inner Mongolia People’s Hospital, Hohhot, China; ^3^ Department of Joint Surgery, The Second Affiliated Hospital, Inner Mongolia Medical University, Hohhot, China; ^4^ Traumatic Orthopedics, Ordos Central Hospital, Ordos, China; ^5^ Inner Mongolia University of Science and Technology, Baotou Medical College, Baotau, China

**Keywords:** Danggui Sini decoction, diabetic foot, network pharmacology, molecular docking, mechanism of action, target

## Abstract

**Objective:**

The main active components and mechanism of Danggui Sini decoction (DSD) in treating diabetic foot (DF) were studied and verified by network pharmacology and molecular docking. Evidence-based medicine was used to prove its efficacy.

**Methods:**

The TCMSP systematic pharmacology platform screened out DSD’s practical components and targets—screening disease targets in GeneCards database, using Cytoscape 3.7.2 to draw DSD–active ingredient–target network diagram, and drawing the protein interaction network diagram through STRING database. The Metascape platform was used to analyze the GO function enrichment and KEGG signal pathway. The molecular docking experiment was carried out by using Auto Dock vina 4.2. The related literature on DSD in treating DF in China Zhiwang, Wanfang, Weipu, and China Biomedical Literature Database was searched. The literature was screened, data was extracted, and quality was evaluated according to the inclusion and exclusion criteria. Then, a meta-analysis was performed using RevMan 5.3 software.

**Results:**

A total of 256 targets of all effective components of DSD were obtained. Among 1,272 disease targets, there are 113 common targets. The GO analysis received 6,179 entries, and the KEGG pathway enrichment analysis found 251 related pathways. The molecular docking results of the main targets of diabetic foot and the active substances of DSD all showed a high docking activity. The meta-analysis included six literature, all of which were randomized controlled experiments. The quality grade of the literature was C, and the results showed that the total effective rate of clinical efficacy in the experimental group was significantly higher than that in the control group.

**Conclusions:**

DSD may treat DF by participating in biological processes such as cell proliferation regulation, inflammatory reaction, oxidative stress reaction, and promotion of angiogenesis. DSD treats DF through AKT1, TP53, IL6, TNF, VEGFA, and other targets. DSD plays a role in treating DF mainly through the AGE-RAGE signaling pathway and PI3K-AKT signaling pathway. The molecular docking results of AKT1, TP53, IL-6, TNF, and VEGFA with the active substances of DSD show that they all have a high docking activity; among them, VEGFA has a higher docking activity. Compared with conventional treatment, DSD has a high effective rate, short wound healing time, large wound healing area, and high ABI index.

## 1 Introduction

Diabetic foot is the most common lower limb complication of diabetic patients (1), and more than 15% of them are at risk of amputation (2, 3). The formation of DF is mainly attributed to diabetes-related peripheral neuropathy and the damage of vascular endothelial cells in distal arteries. Long-term hyperglycemia will cause macrovascular complications and microvascular complications, such as diabetic nephropathy, retinopathy, and DF. With the development of DF, the lesions can gradually progress from the epidermis and down the dermis to the muscles and bones, eventually leading to foot deformity and even lower limb amputation (4–7). In 2023, the International Working Group on the Diabetic Foot (IWGDF) points out that the main methods for treating DF include the following: 1) controlling blood sugar, 2) antibiotic treatment for the infected patients, 3) timely surgical debridement, 4) continuous closed negative pressure drainage, 5) hyperbaric oxygen therapy, and 6) revascularization (8). Other existing treatment schemes include lateral tibial bone metastasis (9), free flap transplantation, arterial bypass transplantation, stem cell transplantation, and so on (10, 11).

Traditional Chinese medicine has a long history of using Chinese medicine to treat DF. DF belongs to the category of “gangrene and boneless gangrene” in Chinese medicine, which refers to the peripheral vascular disease that occurs at the end of limbs and causes gangrene of toe (finger) in severe cases. Chinese medicine believes that the occurrence of this disease is mainly due to unhealthy temper, deficiency of kidney yang, and the invasion of cold pathogens. Article 351 of Selected Readings of Treatise on Febrile Diseases mentioned that DSD is the main prescription for people with cold hands and feet and heartbroken pulse (12). DSD comes from Zhang Zhongjing’s Treatise on Febrile Diseases. This prescription was originally used to treat Jueyin typhoid fever, which was designed for the syndrome caused by the deficiency of blood and cold coagulation of meridians. Chen Yuan, a famous doctor, thinks that this prescription can be used as long as it conforms to the etiology and pathogenesis of blood deficiency and cold coagulation. Although this prescription has been used for more than a thousand years, it has been widely used in clinics because of its rigorous formula, excellent compatibility, excellent efficacy, and no toxic or side effects (13). Modern pharmacological research shows that Danggui Sini decoction has the effects of anticoagulation, antithrombosis, vasodilation, and microcirculation improvement (14–16). It has been used to treat diabetic peripheral neuropathy, diabetic foot, scapulohumeral periarthritis, primary dysmenorrhea, chilblain, and other diseases (17). With the gradual deepening of people’s research on traditional Chinese medicine, DSD has been reported more and more in treating DF. However, the specific mechanism of DSD in treating DF is not clear at present because of its complex and diverse components and extremely complicated interaction with the human body. Therefore, based on network pharmacology and molecular docking, this paper discusses the mechanism of DSD in treating DF, explores its target in treating DF, and verifies its effectiveness by evidence-based medicine.

## 2 Materials and methods

### 2.1 Active components and action targets of DSD

Seven traditional Chinese medicines, “Bai shao, Da zao, Gan cao, Gui zhi, Xi xin, Tong cao, and Dang gui”, were respectively searched by using TCMSP Chinese Medicine System Pharmacology Platform (https://tcmsp-e.com/), and the oral bioavailability (OB) predictive value was >30%, and the drug-likeness predictive value (DL) was >0.18. Then, UniProt protein database (https://www.uniprot.org/) was used to standardize the targets corresponding to all the effective components of DSD, the weight and invalid targets were removed to obtain the drug effect target, then the molecular formula of the active ingredient was searched in PubChem (https://PubChem.ncbi.nlm.nih.gov) database, and the drug-active ingredient-target related data was optimized to generate a visual relational network diagram by using Cytoscape 3.7.2 software.

### 2.2 Acquisition of DF disease targets

The phrase “diabetic foot” was searched in GeneCards database (https://www.genecards.org/) to obtain DF target, and after removing the duplicates, Venny 2.1.0 (https://bioinfogp.cnb.csic.es/tools/Venny/) was used to intersect disease and drug targets to obtain a common target.

### 2.3 Construction of protein–protein interaction network

Using the STRING database (https://string-db.org/), the PPI network analysis of the common drug–disease targets was carried out, and the species was limited to race, with a confidence of 0.4. Other settings were set as the default, the target was transformed to protein, and the protein–protein interaction network diagram of the common target protein was obtained. Then, the abovementioned network data was downloaded in tsv format and imported into Cytoscape to calculate the degree value to screen the sequencing core genes and optimize the PPI network.

### 2.4 GO function enrichment analysis and KEGG signal pathway analysis

Common targets of diseases and drugs were introduced into Metascape (https://metascape.org/gp/index.html#/main/step1), and the species is limited to humans. GO function enrichment analysis and Kyoto Encyclopedia of Genes and Genomes (KEGG) signal pathway analysis were conducted, and the analysis results were evaluated. Using the GO analysis three-in-one histogram and KEGG enrichment bubble diagram obtained by Weishengxin (https://www.bioinformatics.com.cn/), the critical signal pathways were searched on the KEGG platform (Kegg, https://www.kegg.jp/), and the critical target positions were annotated to generate the main signal pathways of DSD in treating DF.

### 2.5 Molecular docking

Three-dimensional molecular structures of seven active components of Danggui Sini Decoction in mol2 format were downloaded from the PubChem database. Then, the water and residues of the active components were removed by using PyMOL software and set as the spatial structure with the lowest binding energy. The human protein UniProtid corresponding to the common target was obtained from the uniprot database (https://www.uniprot.org/), and the 3D structure of the common target was downloaded from the PDB protein database (https://www.rcsb.org/) using UniProt. AutoDock1.5.6 was used to remove protein water, residues, and invalid metal ions, and then the sample was hydrogenated. After that, GridBox was set according to the active pocket of ligand, and vina4.2 was used to dock the active components of DSD with the target protein of the common target. The lowest binding energy and mode were calculated, and the docking results were evaluated. The obtained core compounds are imported into PyMOL software to visualize the 3D structure, and the docking results with the strongest binding ability are obtained. The binding pattern diagram is obtained according to the order of binding energy from small to large.

### 2.6 Meta-analysis

#### 2.6.1 Search strategy

The system searches China HowNet, Wanfang, VIP, and China Biomedical Literature Database, and the retrieval time is from the database establishment to October 2023. Chinese search terms include diabetic foot, diabetic foot ulcer, diabetic gangrene, diabetic lower extremity vascular disease, Danggui Sini decoction, and modified Danggui Sini decoction.

#### 2.6.2 Inclusion and exclusion criteria

The inclusion criteria were as follows: (1) research type, randomized controlled trial; (2) the subjects, regardless of race, age, and sex, were at least diagnosed as one of diabetic foot, diabetic foot gangrene, and diabetic lower extremity vascular disease; DF≥wagner 0; (3) intervention measures: the experimental group was treated with DSD, and the control group was treated with routine treatment; and (4) evaluation indexes: effective rate, average healing time, ankle-brachial index, wound healing area, ankle skin temperature, and at least one index were included in the literature. The exclusion criteria were the following: (1) animal or basic research, (2) literature such as systematic evaluation and review, (3) research that cannot extract complete data, and (4) literature published repeatedly in the same study.

#### 2.6.3 Literature screening, data extraction, and literature quality evaluation

Two researchers independently screened and extracted data, including the first author, intervention measures, disease types, outcome indicators, etc., and then cross-checked these. If the two researchers have different opinions, they will decide after discussing with the third researcher. Cochrane bias risk assessment tool was used to evaluate the quality of randomized controlled trials, including seven aspects, namely: whether to randomly allocate, whether to allocate hidden, whether to use double-masked, whether to use blind method in outcome evaluation, whether to complete outcome data, whether to report selectively, and whether to have other biases. According to the evaluation results, the quality of the literature is classified as follows: grade A is such that there are no high-risk items in all items, which is low bias risk; grade B includes one or two uncertain or high-risk items, which is a moderate bias risk; and grade C includes more than two uncertain or high-risk projects, which is a high bias risk.

#### 2.6.4 Statistical treatment

A meta-analysis was carried out with RevMan5.3 software. The continuous variables were the mean difference (MD) and 95%CI. When the numerical differences were significant OR, the units were different, the standardized mean difference (SMD) was used, and the binary variables were the ratio (OR) and 95%CI. The *Q* test and *I* ^2^ test evaluated the heterogeneity among the studies. When *I* ^2^ was less than 50%, *P* ≥ 0.05, the heterogeneity was small, and the fixed-effect model was selected. If *I* ^2^ ≥50%, *P* < 0.05, the heterogeneity is significant, and the random-effect model is selected. The source of heterogeneity was determined through a sensitivity analysis or only by making a descriptive analysis.

## 3 Results

### 3.1 Screening results of active components of DSD

Based on the platform of TCMSP pharmacology, 13 active components of Bai shao, 29 active components of Da zao, 92 active components of Gan cao, seven active components of Gui zhi, eight active components of Xi xin, four active components of Tong cao, and two active components of Dang gui were retrieved. After eliminating the invalid targets, 109 targets of Bai shao, 394 targets of Da zao, 1,441 targets of Gan cao, 60 targets of Gui zhi, 140 targets of Xi xin, six targets of Tong cao, and 64 targets of Dang gui were obtained, making a total of 2,214 drug targets. The UniProt protein database (https://www.uniprot.org/) was used to standardize the targets corresponding to all the effective components of DSD, and 256 drug targets were obtained after removing the weight and deleting the invalid targets.

### 3.2 DF target acquisition and drug–disease target intersection

**Figure 1:**
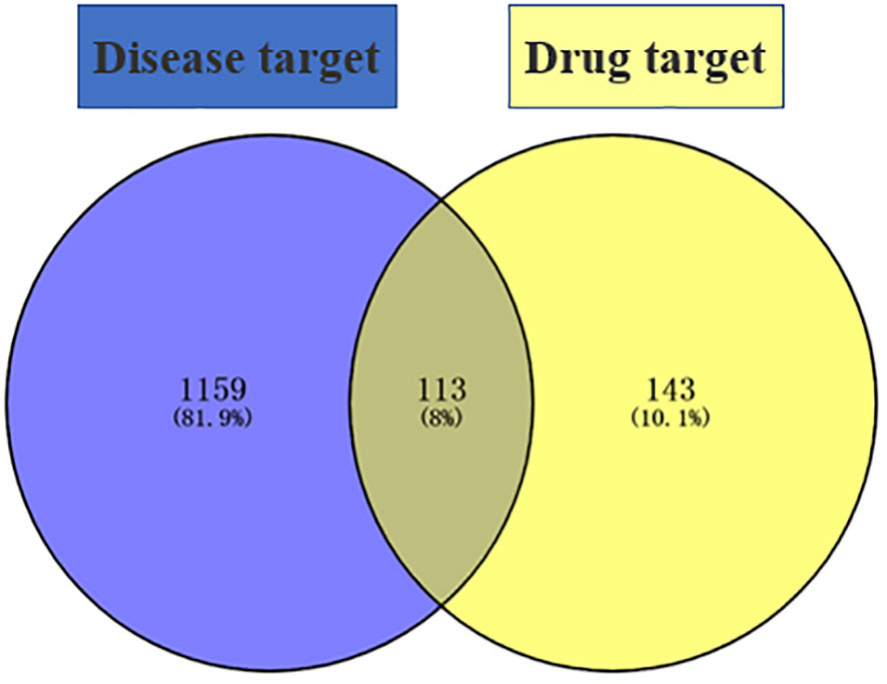
Disease–drug target intersection diagram.

“Diabetic foot” was searched in the GeneCards database (https://www.genecards.org/) to obtain DF targets, eliminating the duplicates to obtain 1,272 disease targets, and Venny 2.1.0 (https://bioinfogp.cnb.csic.es/tools/Venny/) was used to intersect 1,272 disease targets and 256 drug targets to obtain 113 common targets (see **Figure 1**).

### 3.3 Drug–active ingredient–target topological network

**Figure 2:**
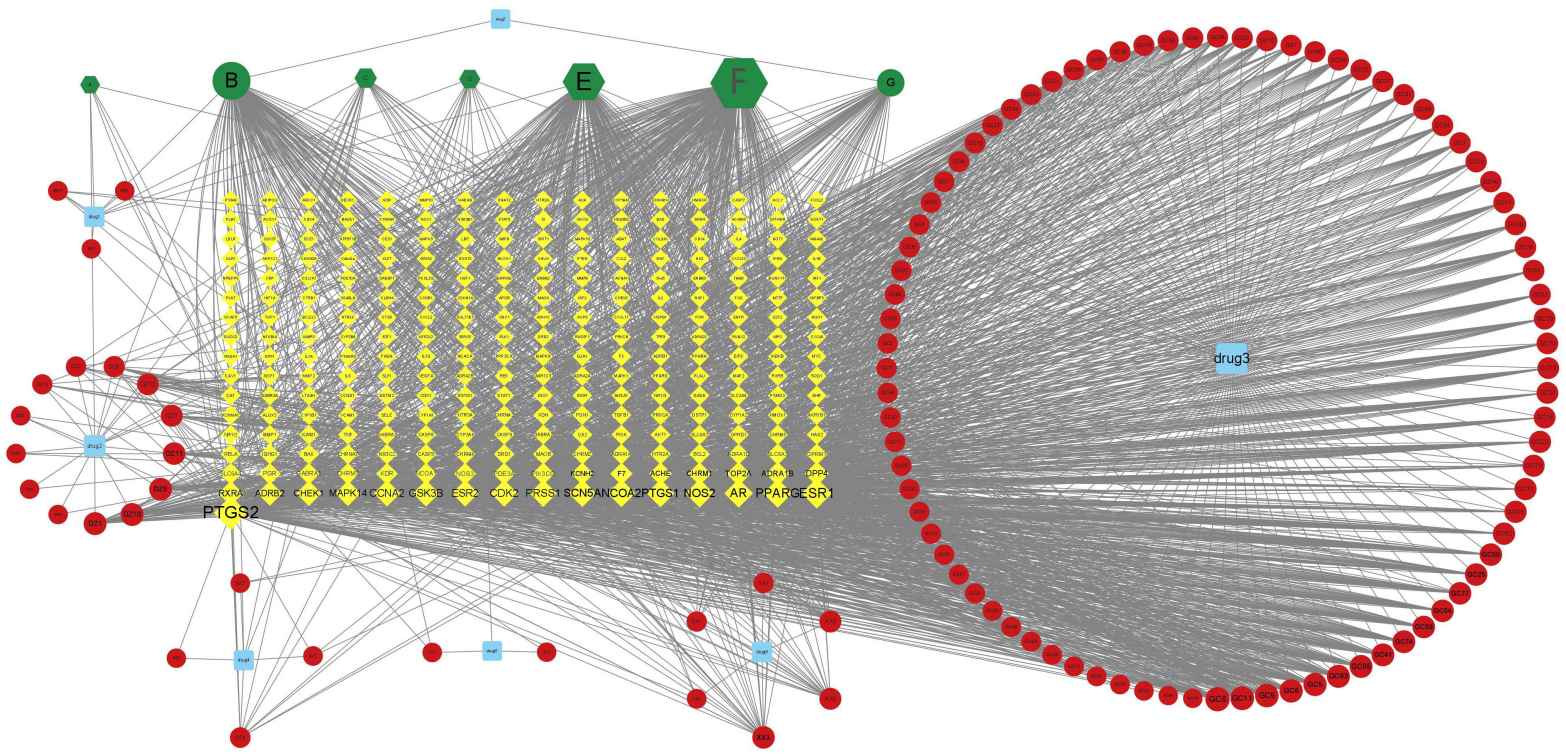
DSD-active component-target topological network diagram.

The drug–active ingredient–target topological network is drawn by using Cytoscape3.7.2 software. The blue squares drug 1–7 respectively represent the seven traditional Chinese medicines of “Bai shao, Da zao, Gan cao, Gui zhi, Xi xin, Tong cao, and Dang gui” in DSD, the green hexagon represents the overlapping active ingredients in the seven medicines (**Table 1**), the red circle represents the active ingredients, and the green circle is for the only two active ingredients of Danggui. From **Figure 2**, it can be seen that the overlapping active ingredients of the three DSD B, E, and F account for the most nodes, so the three active substances, namely, beta-sitosterol, kaempferol, and quercetin, may play an important role in DSD.

**Table 1:** List of the main active components of Danggui Sini decoction in seven traditional Chinese medicines.

MolID molecule name	Active substances overlap drug names			Mark ID	
MOL000211 Mairin	**①**	**②**	**③**		A
MOL000358 beta-sitosterol	**⑦**	**②**	**④**	**⑦**	B
MOL000492 (+)-catechin	**①**	**②**	**④**		C
MOL000359 sitosterol	**④**	**③**	**④**	**⑤**	D
MOL000422 kaempferol	**⑤**	**③**	**⑥**		E
MOL000098 quercetin	**②**	**③**			F
MOL000449 Stigmasterol	**②**	**⑦**			G

**①**, Bai shao; ②, Da zao; ③, Gan cao; ④, Gui zhi; ⑤, Xi xin; ⑥, Tong cao; ⑦, Dang gui.

### 3.4 Construction of protein interaction network

**Figure 3:**
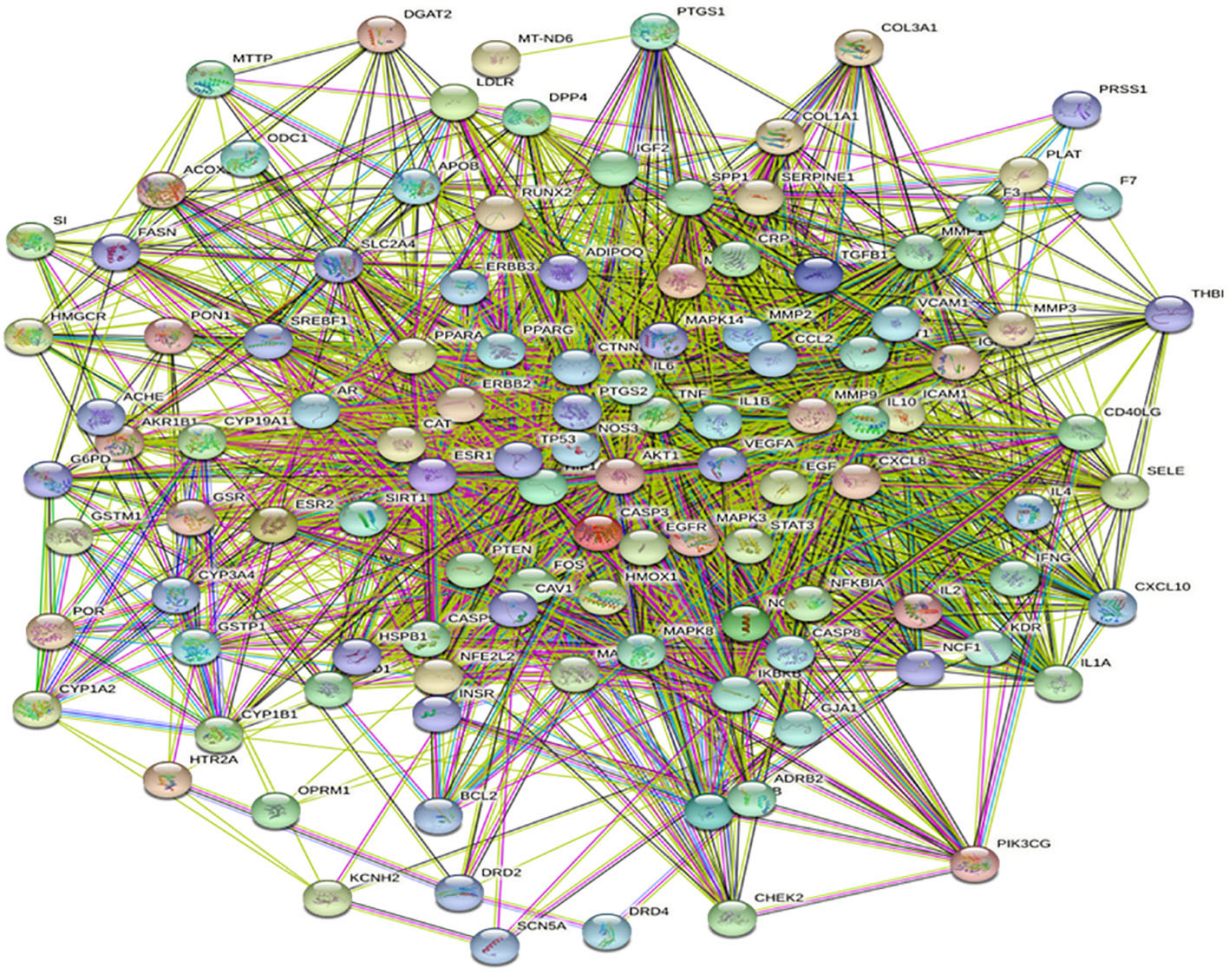
Mutual mapping of common target proteins.

The protein interaction network (PPI) analysis of 113 common targets obtained from the intersection with the STRING database shows that different genes interact with each other, and the interaction between genes is expressed by green, red, purple, and black solid lines, respectively, such as gene proximity, gene estrangement, gene co-occurrence, and gene co-expression. The denser the lines between two nodes, the stronger the correlation between genes, as shown in **Figure 3**.

**Figure 4:**
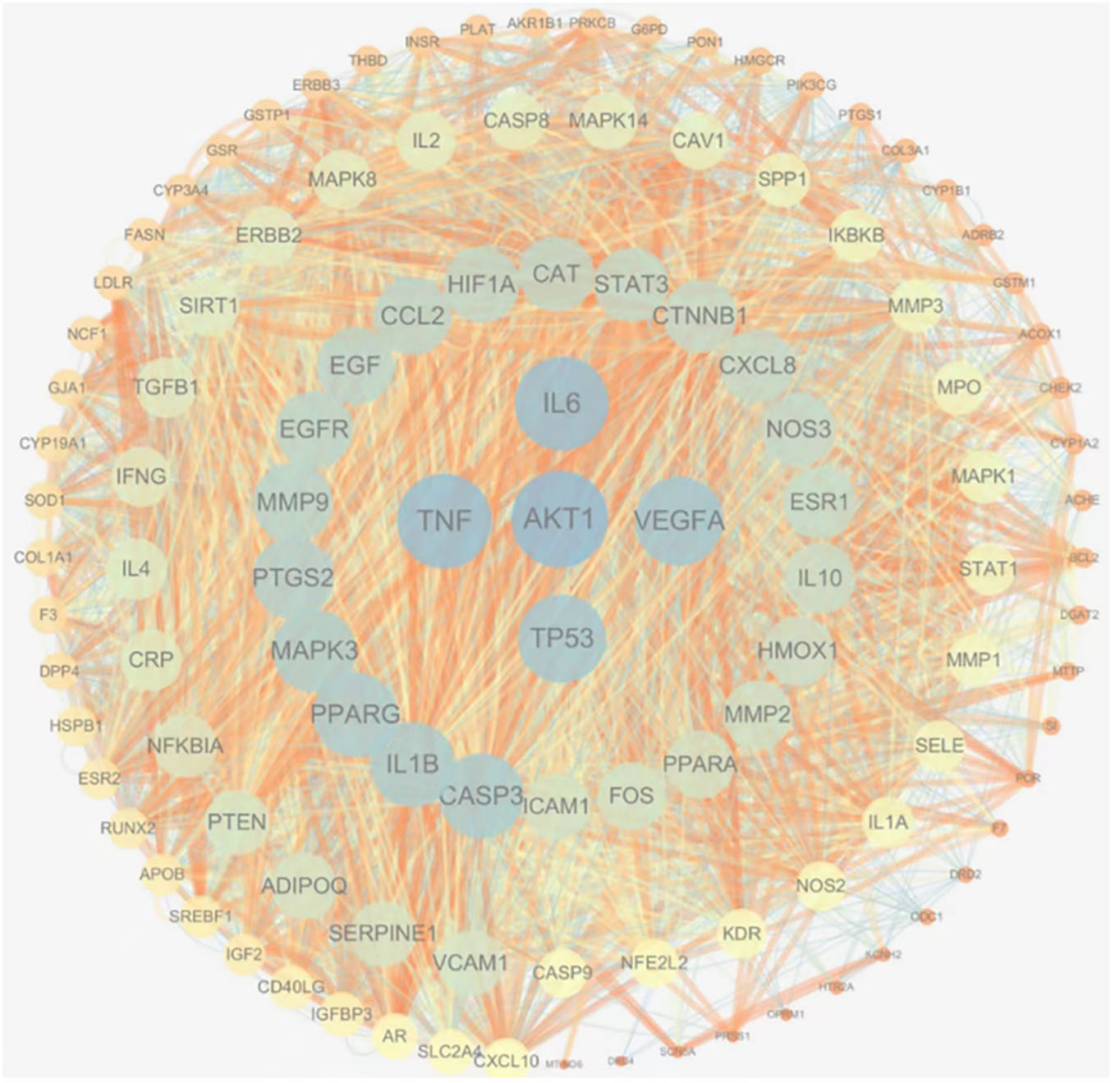
Optimized mutual mapping of the common target protein.

Cytoscape3.7.2 software was used to draw the protein interaction diagram of common targets, in which the size of each node represents its degree, the color from warm to cold represents the centrality from low to high, and the importance of each target is arranged from inside to outside clockwise, as shown in **Figure 4**. It can be intuitively seen from **Figure 4** that targets such as AKT1, TP53, IL6, TNF, and VEGFA play an important role in the network.

### 3.5 GO function enrichment analysis and KEGG signal pathway enrichment analysis

**Figure 5:**
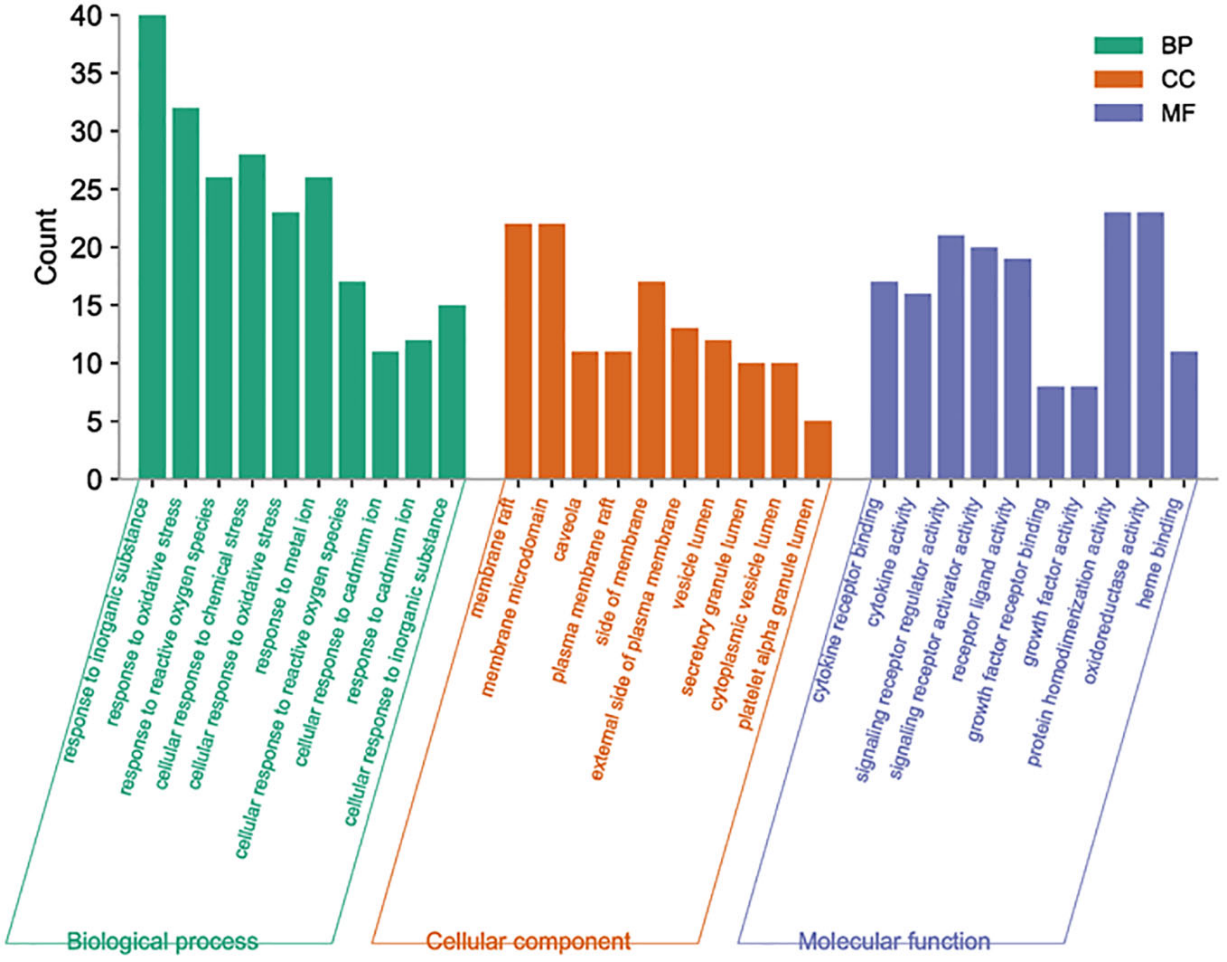
Three-in-one histogram of GO enrichment analysis.

**Figure 6:**
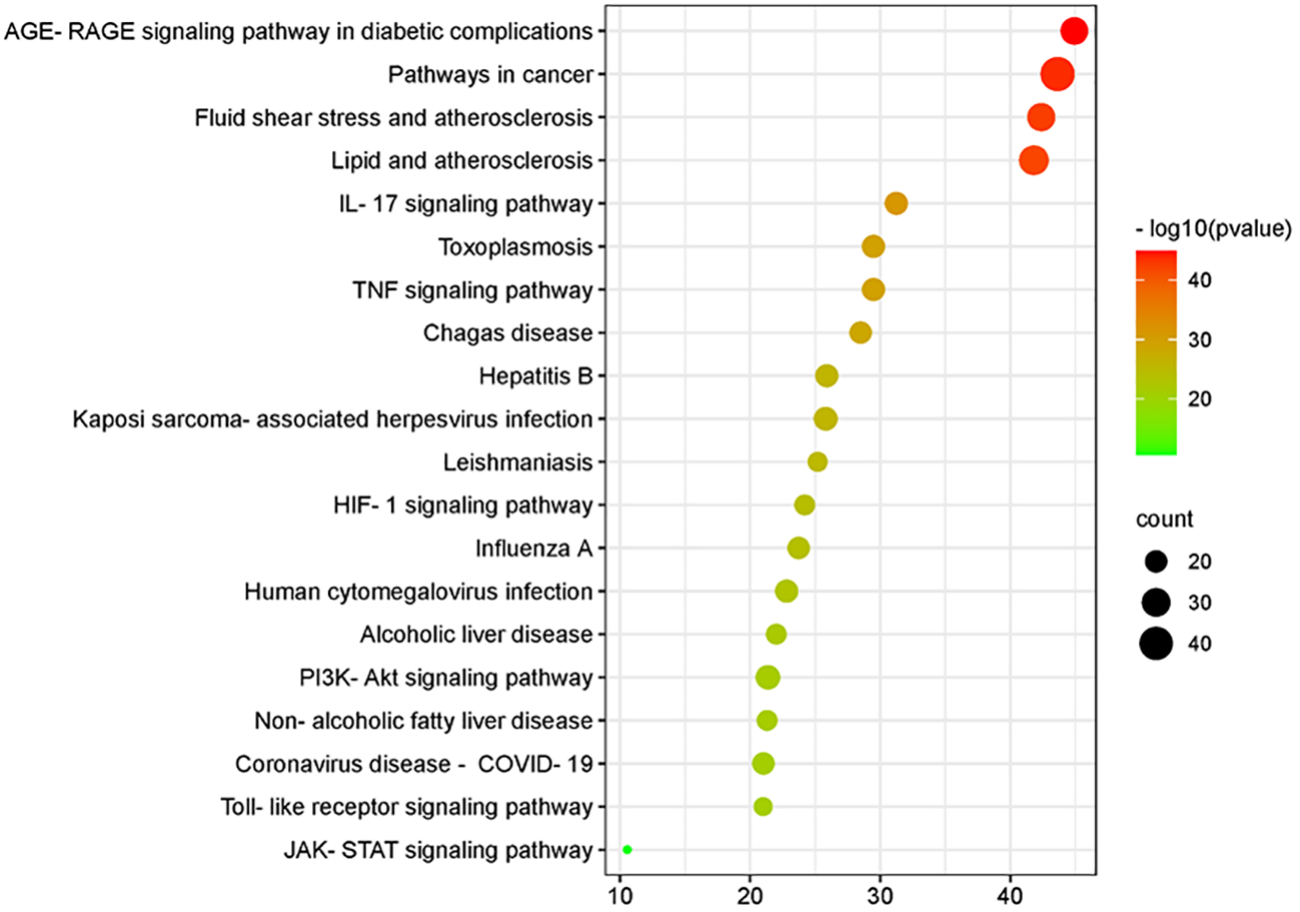
Bubble diagram of the Kyoto Encyclopedia of Genes and Genomes pathway enrichment analysis.

**Figure 7:**
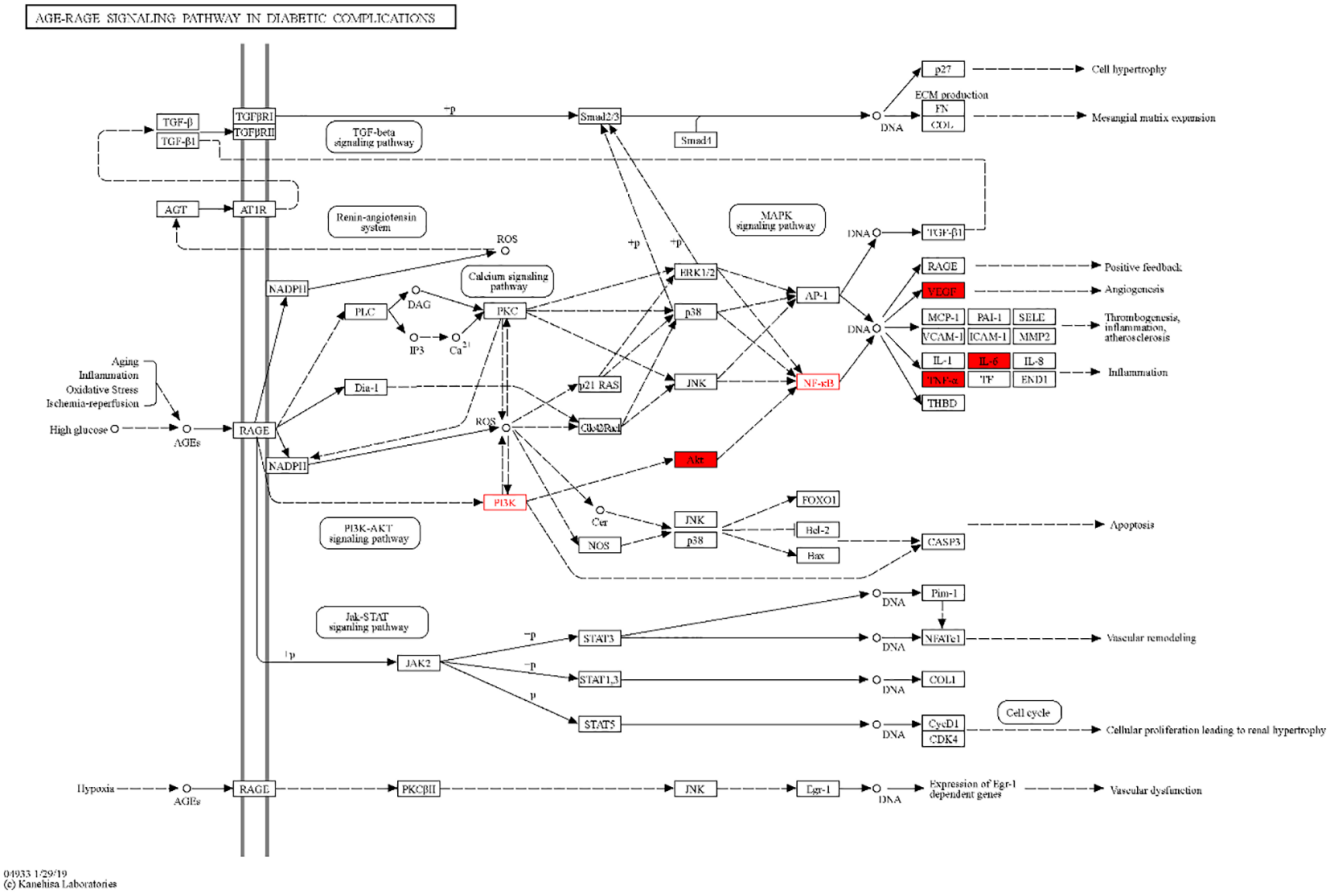
AGEs-RAGE signal pathway.

By analyzing the enrichment of GO function in common target with the Metascape platform, it was found that the main biological processes (BP) mentioned above are the reaction to inorganic substances, the reaction to oxidative stress, and the reaction of cells to chemical stress. Its molecular function (MF) is mainly related to signal receptor regulator and protein homodimerization activity oxidoreductase activity. Cell components (CC) are mainly in the membrane raft, membrane micro-area, and membrane side; the KEGG pathway enrichment analysis found 251 related pathways, and the top 20 most important signal pathways were selected according to the number of pathways involved, and the bubble diagram of KEGG pathway enrichment analysis was drawn. The most important pathways include the AGE-RAGE pathway, cancer-related pathway, fluid shear stress and atherosclerosis pathway, IL-17 signaling pathway, TNF signaling pathway, HIF-1 signaling pathway, Toll-like receptor signaling pathway, PI3K-AKT signaling pathway, P53 signaling pathway, and so on. The KEGG enrichment bubble diagram and BP, CC, MF three-in-one histogram were drawn using Weishengxin (https://www.bioinformatics.com.cn/). The analysis of the mapped signal pathways shows that the AGE-RAGE pathway plays a key role (see **Figures 5**, **6**). Then, the key signal pathways were searched on the KEGG platform (https://www.kegg.jp/, Kegg), and the main signal pathways of DSD in treating DF were generated, as shown in **Figure 7**. It can be seen from **Figure 7** that the active components of DSD may activate the PI3K-Akt signaling pathway through the AGEs-Rages signaling pathway to promote AKT1 production and then further produce NF-κB activity, reduce the expression of inflammatory cytokines (such as IL-8, IL-6 and TNF-α), and promote the expression of atherosclerosis-related genes VEGF and RAGE, thus alleviating inflammatory reaction, promoting angiogenesis, and treating DF.

### 3.6 Molecular docking result

**Figure 8:**
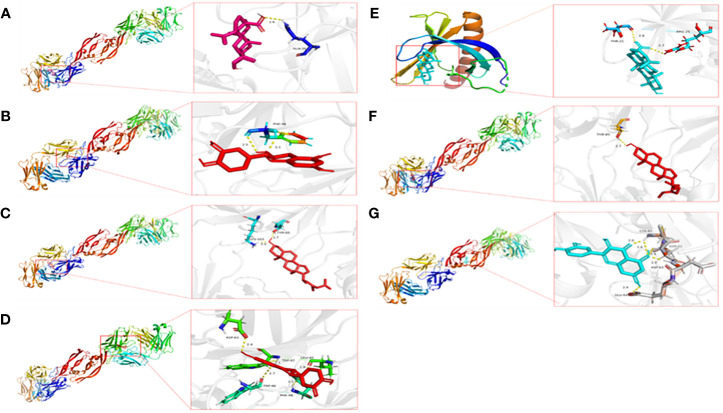
Pattern diagram of the molecular docking results. **(A)** VEGFA and Mairin, **(B)** VEGFA and kaempferol, **(C)** VEGFA and stigmasterol, **(D)** VEGFA and (+)-catechin, **(E)** AKT1 and Mairin, **(F)** VEGFA and beta-sitosterol, **(G)** VEGFA and quercetin.

The active components of drugs are regarded as ligands, and the proteins encoded by the common target are regarded as receptors. Then, the ligands corresponding to the common target are selected from the drug–active component–target topological network for AutoDock Vina docking, and each complex’s binding energy and binding mode are calculated. Finally, docking was carried out 35 times, and the docking results are shown in **Table 2**. The first seven binding energies are taken to get the binding pattern in **Figure 8**.

**Table 2:** Docking table of the active components and common target protein of Danggui Sini decoction.

Receptor and ligand	Species	PDB ID	Binding energy(kcal/mol)
VEGFA&Mairin	HSP	1BJ1	-8.1
VEGFA&kaempferol	HSP	1BJ1	-7.4
VEGFA&stigmasterol	HSP	1BJ1	-7.4
VEGFA&(+)-catechin	HSP	1BJ1	-7.2
AKT1&Mairin	HSP	2UZR	-7.2
VEGFA&beta-sitosterol	HSP	1BJ1	-7.1
VEGFA&quercetin	HSP	1BJ1	-7.1
TNF&beta-sitosterol	HSP	1TNF	-6.9
TP53&stigmasterol	HSP	1A1U	-6.8
IL6&Mairin	HSP	1ALU	-6.8
AKT1&(+)-catechin	HSP	2UZR	-6.8
AKT1&kaempferol	HSP	2UZR	-6.7
IL6&beta-sitosterol	HSP	1ALU	-6.7
IL6&stigmasterol	HSP	1ALU	-6.7
AKT1&quercetin	HSP	2UZR	-6.6
TNF&stigmasterol	HSP	1TNF	-6.6
TP53&beta-sitosterol	HSP	1A1U	-6.6
AKT1&stigmasterol	HSP	2UZR	-6.5
TNF&Mairin	HSP	1TNF	-6.5
TP53&Mairin	HSP	1A1U	-6.5
IL6&(+)-catechin	HSP	1ALU	-6.4
IL6&kaempferol	HSP	1ALU	-6.4
AKT1&beta-sitosterol	HSP	2UZR	-6.3
IL6&quercetin	HSP	1ALU	-6.3
TNF&kaempferol	HSP	1TNF	-6.2
TNF&quercetin	HSP	1TNF	-6.1
TP53&quercetin	HSP	1A1U	-6.0
TNF&(+)-catechin	HSP	1TNF	-5.9
TP53&(+)-catechin	HSP	1A1U	-5.7
TP53&kaempferol	HSP	1A1U	-5.7

Usually, the binding energy is less than -8 kcal/mol, meaning that the receptor has a strong free-binding ability with the ligand. A binding energy of less than -5.5 kcal/mol indicates that the free binding ability is strong. A binding energy of less than 4.25 indicates that the binding capacity is average. The docking results show that the binding capacity of all ligands and receptors is less than -5.5 kcal/mol. The binding capacity of VEGFA and Mairin is strong, among which the binding capacity of VEGFA and Mairin is less than -8 kcal/mol and six receptors in the top 7 ligand-receptor combinations are VEGFA, and the other one is AKT1, which has a strong binding capacity with Mairin. VEGFA is hydrogen-bonded to GLN at the 39th site of Mairin protein, PHE at the 98th site of kaempferol protein, and THR and LYS at the 85th and 103rd sites of stigmasterol. It is bonded with LEU, TRP, ASP, TRP, and PHE at sites 45, 47, 63, 96, and 98 of (+)-catechin, with THR at site 85 of beta-sitosterol, and with CYS, ASN, ASP and 64 at sites 61, 62, 63, and 64 of quercetin. Molecular docking research shows that the active components and targets in Danggui Sini decoction may bind to the target protein through the abovementioned binding mode, thus playing a role in treating DF.

### 3.7 Meta-analysis results

#### 3.7.1 Literature retrieval results, basic characteristics, and quality of included documents

**Figure 9:**
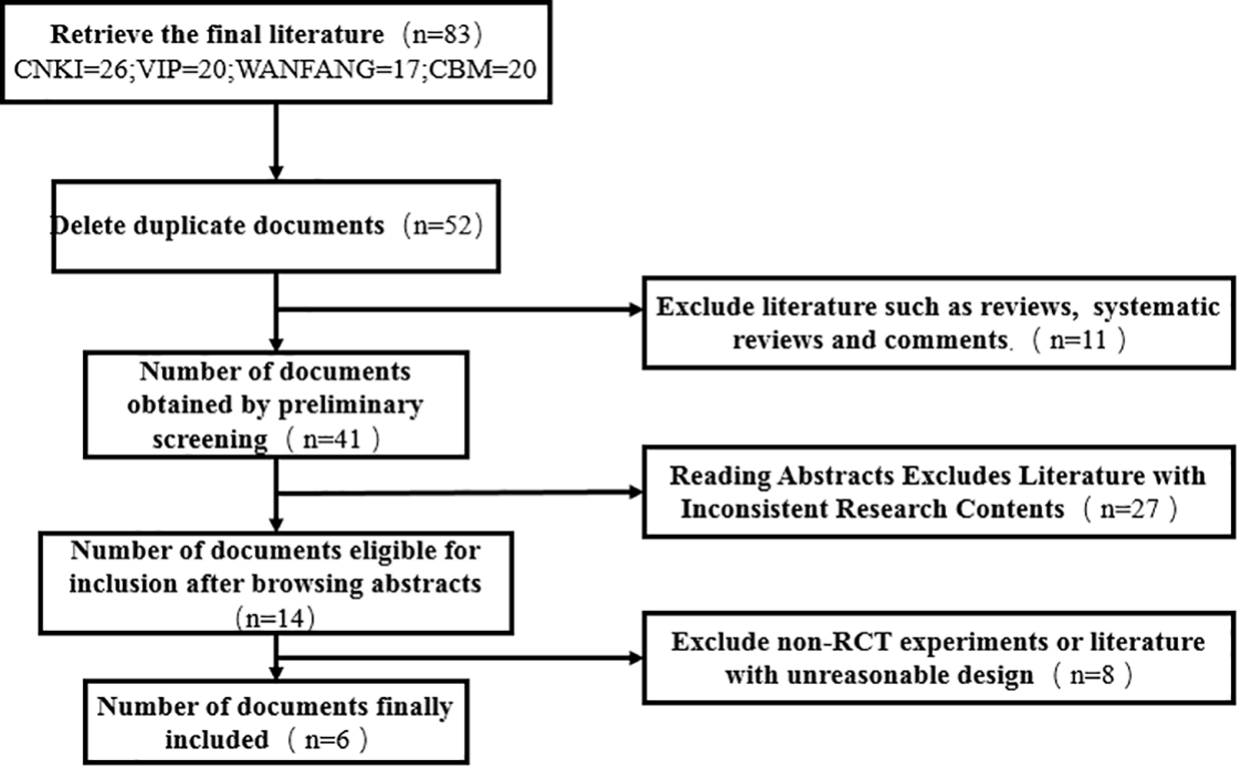
Screening process.

**Figure 10:**
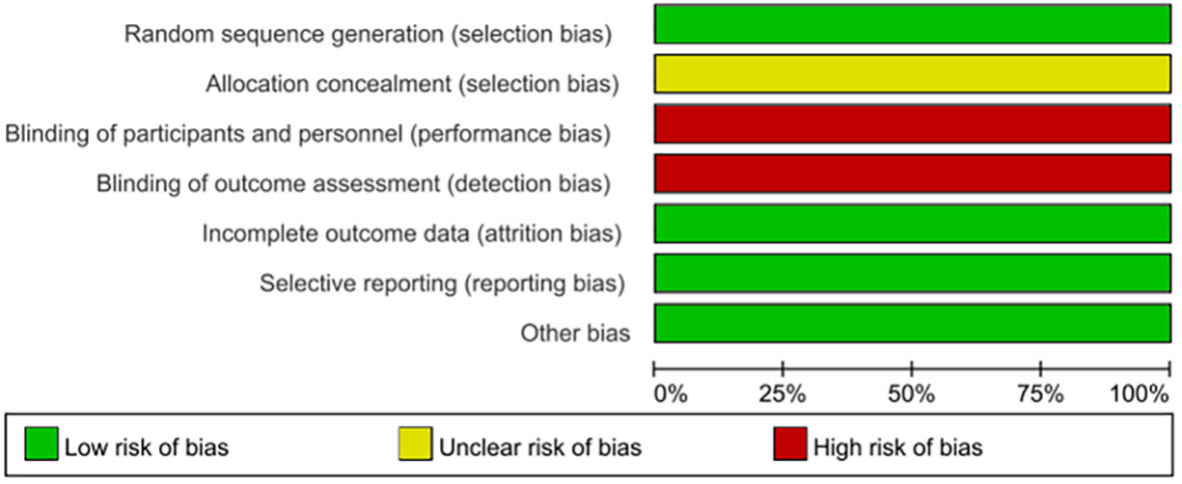
Incorporates a risk bias map.

As DSD belongs to traditional Chinese medicine, there is no similar research abroad, so only domestic literature was searched—83 literatures were retrieved according to the retrieval strategy, and six literatures were selected strictly according to the inclusion and exclusion criteria (18–23), with a total of 444 patients (224 cases in the experimental group and 220 cases in the control group), all of which were randomized controlled trials (see **Figure 9** for the flow chart of literature screening; see **Table 3** for the basic features of the included documents). The quality evaluation results of the randomized controlled trials are shown in **Figure 10**, respectively. Because of the particularity of traditional Chinese medicine treatment, it is impossible to carry out blind experiments, so the quality grade of all articles is C.

**Table 3:** Basic characteristics of the included documents.

Author	year	Method	Total sample	Gender	Age (years)	Course of DF	Intervention measure	Outcome index
			Experiment/control	Group (male/female)	Group (mean/mean standard deviation)	Group (mean/mean standard deviation)		
Yuan (24)	2010	RCT	32/28	(17/15)/(14/14)	57.5/58.5	4.5 year/5.0 year	DSD	①
Cao (25)	2015	RCT	30/30	(14/16)/(13/17)	54.12 ± 8.16/52.78 ± 9.18	3 months–8 years/2 months–8 years	DSD	①, ②, ⑤
Chen (26)	2022	RCT	49/49	(30/19)/(26/23)	66.85 ± 2.95/67.18 ± 2.74	11.45 ± 3.15 months/10.83 ± 2.35 months	A+DSD/A	①,②,③,⑥
Yu (27)	2023	RCT	58/58	(35/23)/(32/26)	51.64 ± 2.71/52.37 ± 2.84	14.46 ± 3.21 months/15.22 ± 3.1 7months	B+DSD/B	①,③,⑦
Fan (28)	2023	RCT	20/20	(10/10)/(10/10)	51.2 ± 22.5/53.1 ± 24.3	Unspecified	C+DSD/C	③,⑧
Qi (29)	2023	RCT	35/35	(19/16)/(18/17)	58.80 ± 7.615/59.23 ± 7.337	12.00 ± 6.73 months/10.49 ± 5.76 months	D+DSD/D	①,②,④,⑨

A, fumigation and washing with traditional Chinese medicine; B, acupuncture treatment; C, transverse bone transfer of tibia; D, Beraprost sodium; ①, efficiency; ②, ankle-brachial index; ③, wound healing; ④, color Kepler ultrasound integral of arteries of both lower limbs; ⑤, sensory threshold; ⑥, hemodynamic index, nerve conduction velocity; ⑦, IL-1, IL-6, TNF-α; ⑧, lower limb ischemia index; ⑨, blood sugar and blood lipid.

#### 3.7.2 Meta-analysis results

**Figure 11:**
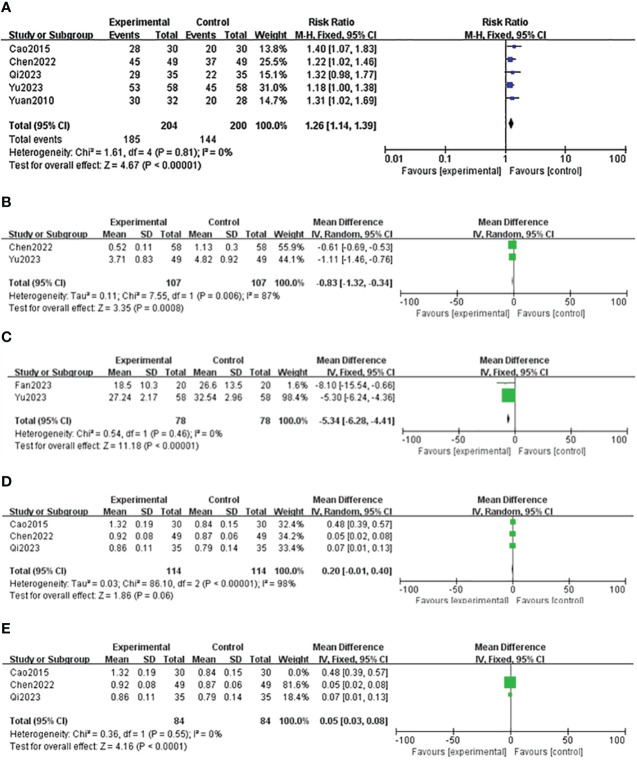
Forest map of the effective rate of Danggui Sini decoction (DSD) in treating diabetic foot (DF). **(A)** Effective rate of DSD in treating DF. **(B)** Wound healing area of DSD in treating DF. **(C)** Wound healing time of DSD in treating DF. **(D)** ABI index of patients with DF. **(E)** ABI index of patients with DF after eliminating bias.

**Figure 12:**
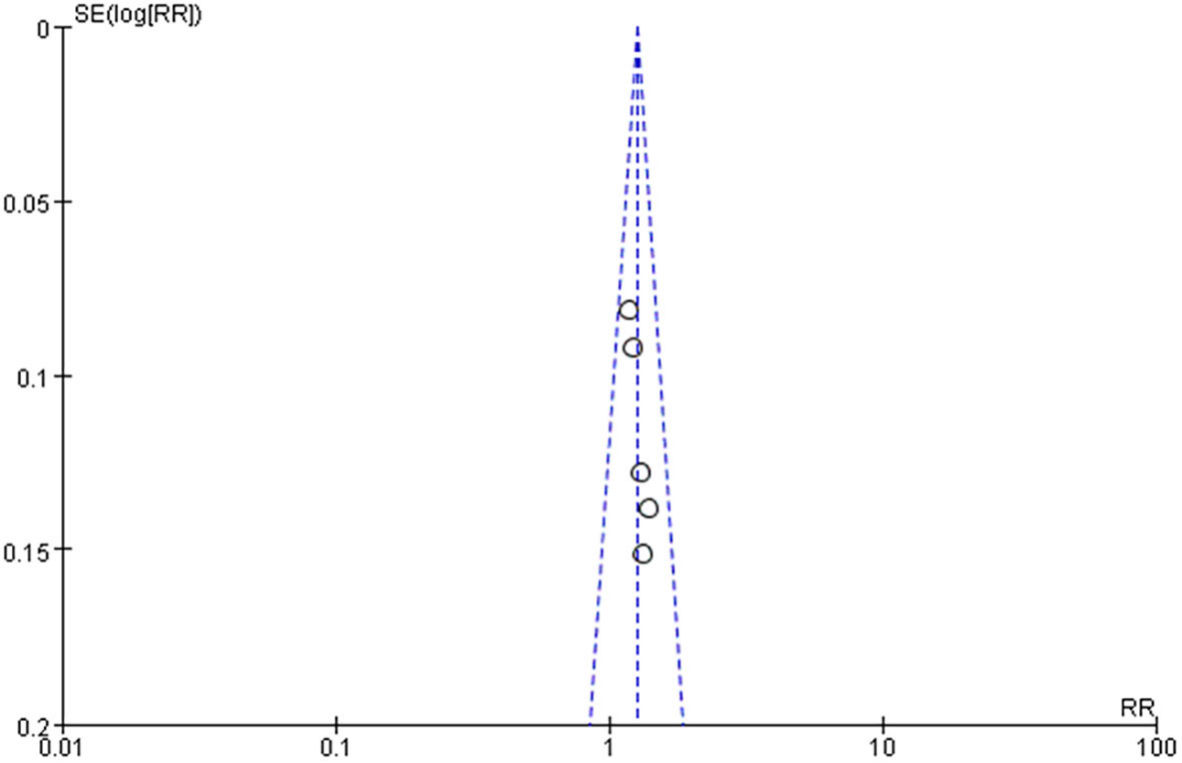
Funnel diagram of the effectivity rate of Danggui Sini decoction in treating diabetic foot.

Five studies reported the effective rate of DSD in treating DF (24–27, 29), and the heterogeneity among the study groups was low (*I* ^2 =^ 0 <50%, *P* = 0.81 ≥ 0.05). Therefore, the fixed-effect model was used for statistical analysis. The results showed that the effective rate of the experimental group was higher than that of the control group (RR = 1.26, 95% CI: 1.14–1.39, *P* < 0.00001), as shown in **Table 4** and **Figure 11A**. Two studies reported the wound healing area of DSD in treating DF (26, 27), (*I* ^2 =^ 87% > 50%, *P* = 0.006 < 0.00001). The random-effect model analyzed the data, and the results showed that the wound healing area of the experimental group was higher than that of the control group (MD = -0.83, 95%CI: -1.32–0.34, *P* = 0.0008), as shown in **Table 4** and **Figure 11B**. Two studies reported the wound healing time of DSD in treating DF (27, 28), and the heterogeneity between the study groups was low (*I* ^2 =^ 0 <50%, *P* = 0.46 ≥ 0.05). Thus, the fixed-effect model was used for statistical analysis, and the results showed that the wound healing time of the experimental group was lower than that of the control group (MD = -5.34, 95%CI: -6.28–4.41, *P* < 0.00001), as shown in **Table 4** and **Figure 11C**. Three studies reported the ankle-brachial index (25, 26, 29) of DSD in treating DF, and the heterogeneity among the study groups was high (*I* ^2 =^ 98% > 50%, *P* = < 0.00001) (MD = 0.20, 95%CI: -0.01–0.40, *P* = 0.06 > 0.05) The results showed no statistical significance, as shown in **Table 4** and **Figure 11D**. The sensitivity analysis of the ankle-brachial index shows that the heterogeneity is obviously reduced (*I* ^2 =^ 0, *P* = 0.55, MD = 0.05, 95% CI: 0.03–0.08, *P* < 0.0001) after excluding the research of Cao Li et al. It has been proved that the ankle-brachial index of the experimental group is better than that of the control group, as shown in **Table 4** and **Figure 11E**. The sensitivity analysis of the effective rate shows that the research results have not changed significantly, suggesting that the results are reliable and stable, and the rest of the outcome indicators are only included in two documents, so only a descriptive analysis was made. Taking the effective rate of the outcome index with the most references as an example to evaluate the publication bias, it is suggested that the publication bias is small, as shown in **Figure 12**.

**Table 4:** Comparison between Danggui Sini decoction and conventional therapy for diabetic foot.

Outcome index	Incorporate literature	RR/MD	95%CI	*P*	Heterogeneousness		Effect model
					*I*²	*P*	
Efficiency	19–22, 24	RR = 1.26	1.14–1.39	<0.00001	0	0.81	Fixed effect
ABI	21, 24	MD = 0.05	0.03–0.08	<0.0001	0	0.55	Fixed effect
Wound healing area	21, 22	MD = -0.83	-1.32–0.34	0.0008	87%	0.06	Random effect
Wound healing time	22, 23	MD = -5.34	-6.28–4.41	<0.00001	0	0.46	Fixed effect

## 4 Discussion

DF is a common complication of long-term diabetes, which is related to the high incidence of lower limb ulcers and amputations. There are many reasons for DF. When venous ischemia or a bacterial infection occurs in the lower limbs, it often leads to coldness in both lower limbs, pain in both limbs, and even ulcerative rot. At present, DF is mainly treated by anti-infection and limb debridement. However, when diabetic patients have infected wounds, the wounds are large and slow to heal, leading to poor prognosis (30). Therefore, promoting the healing of diabetic refractory skin ulcer and reducing the disability rate are the research hotspots. Several ancient prescriptions in traditional Chinese medicine have been used to treat DF. DSD, as a traditional Chinese medicine prescription, has a unique effect on anti-inflammation, anticoagulation, promoting angiogenesis, and inhibiting AGE production (31), but its specific effect is still unclear. Therefore, this study used the method of network pharmacology to explore the key active components of DSD in treating DF.

The research results of network pharmacology show that eugenol, β-sitosterol, (+)-catechin, kaempferol, quercetin, and coumarol are important components of DSD, and they have many targets that intersect with the disease targets of DF. These compounds have a wide range of anti-inflammatory, immunomodulatory, anti-tumor, and anti-oxidation effects, such as Jayachandran M et al. (32) who found the level of lipid peroxide in diabetic rats. In addition, the inflammatory reaction can be inhibited by regulating the polarization conversion of macrophages from the M1 phenotype to the M2 phenotype (18). Moreover, sitosterol and stigmasterol also have anti-inflammatory effects (19).

The PPI network and topological analysis show five main targets for active ingredients in DSD, and AKT1 is now considered a mature and vital target for treating DF. According to related studies, AKT1 can regulate the proliferation of vascular endothelial cells (20), fibroblast proliferation (21), and postprandial blood glucose level (22) through the downstream mTOR/eNOS and reduce the release of inflammatory factors through its downstream IKK/NF-κB pathway. In addition, PI3K/AKT can reduce the apoptosis of islet β cells, while AKT/GSK3β can promote the regeneration of islet β cells (33), and IL-6 and TNF-α are proinflammatory factors. The research of Chesworth et al. shows that the IL-6 and TNF-α levels increase during infection or tissue injury, and their low expression levels are of great significance to DF wound healing (34). VEGF is considered the most potent angiogenic factor, which can promote the growth of endothelial cells through vascular endothelial growth factor receptors. VEGF drugs are often used to treat foot ulcers and are essential in wound healing (35, 36).

The molecular docking results show that all DSD’s active components have the best docking effect with vascular endothelial growth factor (VEGF), and DSD may promote wound healing and treat DF by regulating VEGF. The target protein of DSD in the treatment of DF may be VEGF, which can further cause changes in serum IL-6 and TNF-α levels, reduce inflammatory response, and play an anti-inflammatory role. The AGE-RAGE signaling pathway and PI3K-AKT signaling pathway may mediate these effects.

The meta-analysis results show that DSD is superior to the control group in the effective rate, ankle-brachial index, wound healing area, and wound healing time. At the same time, the research of Yu Yanhua (27) shows that DSD can regulate nerve conduction velocity, inhibit the percentage of IL-1, TNF-α, IL-6, and neutrophils, and reduce inflammatory reactions. Cao Li et al. (25) showed that DSD can improve the sensory threshold and relieve patients’ pain effectively. Fan Zhiqiang et al. (28) showed that DSD combined with lateral tibial bone transfer guided by a 3D guide plate was used to improve the local microcirculation of DF to treat diabetic foot ulcer and avoid amputation. The research of Qi Yunping et al. (29) shows that DSD can improve DF patients’ blood sugar and blood lipid levels.

## 5 Conclusion

In a word, this study preliminarily explored the mechanism of DSD on DF through network pharmacology and molecular docking technology in order to verify that the potential mechanism of DSD in treating DF may be related to the AGEs-RAGE signaling pathway and PI3K-AKT signaling pathway, reducing inflammatory reaction and increasing VEGF expression. It shows that DSD has the hierarchical network characteristics of “multi-component, multi-target, multi-function, and multi-channel” in the treatment of DF, which provides a basis for further clarifying the effective target of drugs. The meta-analysis summarized six randomized controlled experiments, and it was concluded that DSD had the advantages of high efficiency, short wound healing time, large wound healing area, and improvement of the ankle-brachial index. It could also improve the blood sugar and blood lipid indexes of diabetic patients, increase nerve conduction speed, improve microcirculation, inhibit inflammatory factors, and reduce amputation rate. However, there still needs to be more animal, cell, and molecular biology experiments to verify the target and pathway of DSD objectively in treating DF from the aspects of genes, proteins, tissues, and organs.

## Data availability statement

The original contributions presented in the study are included in the article/supplementary material. Further inquiries can be directed to the corresponding author.

## Ethics statement

Ethical review and approval was not required for the study on human participants in accordance with the local legislation and institutional requirements. Written informed consent from the patients/participants or patients/participants’ legal guardian/next of kin was not required to participate in this study in accordance with the national legislation and the institutional requirements.

## Author contributions

XN: Conceptualization, Investigation, Software, Writing – original draft, Writing – review & editing. HB: Project administration, Supervision, Writing – review & editing. JG: Data curation, Formal analysis, Methodology, Writing – original draft, Writing – review & editing. LD: Conceptualization, Investigation, Software, Writing – original draft, Writing – review & editing. LW: Data curation, Writing – original draft. WZ: Writing – original draft. GS: Funding acquisition, Project administration, Resources, Writing – review & editing.
